# Experience Alone Can Generate Human Face Specialization: Evidence From Deep Learning Models

**DOI:** 10.1162/OPMI.a.363

**Published:** 2026-07-07

**Authors:** Nitzan Guy, Mandy Rosemblaum, Galit Yovel

**Affiliations:** School of Psychological Sciences, Tel Aviv University, Tel Aviv, Israel; Sagol School of Brain Sciences, Tel Aviv University, Tel Aviv, Israel

**Keywords:** developmental, inversion, other race, neural networks

## Abstract

The specialization of human face recognition for upright own-race faces is well-established. While experience is thought to play a key role in face specialization, establishing its direct causal contribution in humans is difficult, as natural experience cannot be systematically controlled. Recent advances in deep learning algorithms offer a solution: these algorithms were shown to generate human-like face specialization effects, including the face inversion and other-race effects. Critically, deep neural networks allow precise manipulation of their training experience, allowing us to test its sole contribution to human-like face expertise in artificial systems. In the present study, we systematically manipulated the amount of face experience provided to deep neural networks and examined its effect on the face inversion, the other-race and other-age effects. Mirroring human development, the magnitude of the other-group and face inversion effects increased with greater own-group upright face experience. These effects were primarily driven by a steep improvement in recognition of upright, own-group faces, with much shallower gains for other-group or inverted faces. These findings demonstrate that increased exposure to upright own-group faces selectively improves performance for this category, establishing that experience alone is sufficient to produce human-like face specialization effects in artificial systems.

## INTRODUCTION

Face recognition is a fundamental cognitive and social ability, supported by dedicated cognitive and neural mechanisms. This ability is further characterized by a specialization for upright, own-group faces. This specialization is evident in robust behavioral effects, including the well-established face inversion effect—where recognition performance is significantly impaired for inverted compared to upright faces (Farah et al., 1995; Valentine, 1988; Yin, 1969)—and in the enhanced recognition of faces from one’s own race or age group, known as the other-race (Meissner & Brigham, 2001; Rossion & Michel, 2011) or other-age effects (Rhodes & Anastasi, 2012) or more generally the other-group effect. These face specialization effects are thought to emerge from the extensive experience humans have with upright own-group faces throughout their life (McKone et al., 2019; Rhodes et al., 2010). However, several additional factors have been proposed to account for these face specializations, including innate predispositions (Johnson, 2005; Pascalis & Kelly, 2009), holistic processing mechanisms (Farah et al., 1995; Hugenberg & Corneille, 2009; Michel, Caldara, & Rossion, 2006; Michel, Rossion, et al., 2006; Rhodes et al., 2006), levels of categorization (Gordon & Tanaka, 2011; Levin, 1996; Schwartz & Yovel, 2016; Yovel et al., 2012), and social and motivation influences (Bernstein et al., 2007; Hugenberg et al., 2010; Young & Hugenberg, 2012). Disentangling the contributions of these different factors in humans is challenging, as they cannot be independently manipulated. The present study aims to isolate the role of experience with faces by assessing its sole contribution to face specialization using computational modeling.

Evidence that experience shapes face recognition abilities is abundant. The role of experience in the other race effect is demonstrated in studies using face recognition tasks that show that early experience with faces of the other race abolishes or reverse the other-race effect (de Heering et al., 2010; McKone et al., 2019; Sangrigoli et al., 2005). In particular, studies that examined face recognition in individuals of Asian descent who were adopted by European parents at a young age and raised primarily among White individuals found that these adoptees exhibited either superior performance in old/new tasks for White faces compared to Asian faces (Sangrigoli et al., 2005) or no difference between the two race groups (de Heering et al., 2010). Moreover, a recent study suggested that there might be a critical period during which other-race faces can reach performance level as own race faces. This study indicated that exposure to other-race faces after the age of 12 does not abolish the other-race effect (McKone et al., 2019). These findings highlight the central role of early and sustained exposure in shaping face recognition capabilities across racial categories.

A related phenomenon that further underscores the importance of experiential factors is the other-age effect, which refers to enhanced recognition performance for faces belonging to the age group with which individuals predominantly interact. This effect has been consistently observed in face recognition tasks showing diminished identity recognition accuracy for children’s or infants’ faces by adults (Kuefner et al., 2008; Yovel et al., 2012). It has been further shown that teachers who work with children do not show this other-age effect (Harrison & Hole, 2009), again indicating that experience with a certain category of faces improves performance on face identity recognition of that age category. Interestingly, passive exposure to faces from an other-age which does not involve individuation does not improve face recognition, indicating that it is the act of identification rather than perceptual exposure to faces per se that is critical for face recognition (Yovel et al., 2012).

The role of experience is further evidenced by the improvement in face recognition abilities during development. Studies have shown that performance on face identity recognition across varied head orientation continues to improve after the age of 10 (Mondloch et al., 2003). Moreover, improvement in recognizing identities in memory tasks (old/new and Cambridge Face Memory Test (CFMT)) continues even after the age of 30 (Germine et al., 2011). These studies suggested that experience continues to play an important role in face recognition even after the early stages of development through adulthood. The developmental enhancement was also reported for the face inversion effect. Whereas preference for looking at upright relative to inverted faces was shown shortly after birth (Viola Macchi et al., 2004), the magnitude of the face inversion effect substantially increases across development. This increase primarily results from improved performance for upright faces, with little to no improvement for inverted faces (de Heering et al., 2012; Lewis & Hills, 2018; Megreya & Bindemann, 2015; Meinhardt-Injac et al., 2014). These findings suggest that with development, the face recognition system becomes increasingly fine-tuned to upright faces, while recognition performance for inverted faces remains static. Consistent with this experience-based account, a recent case study of an individual born with his head oriented upside down revealed superior face detection and identity recognition for inverted faces compared to upright faces (Duchaine et al., 2023), demonstrating that lifelong experience viewing faces from an inverted perspective can reverse the typical face orientation advantage.

The developmental trajectory of the other-race effect has also been examined. While several studies report an increase in the magnitude of the other-race effect with age, others have found no significant developmental differences in the strength of this effect (Anzures et al., 2013; Chance et al., 1982; Goodman et al., 2007). These mixed findings underscore the complexity of developmental changes in face recognition and the influence of experiential factors in shaping performance across different face categories.

Taken together, developmental studies indicate that both the face inversion effect and the other-race effect become increasingly adult-like with age, highlighting the role of experience with upright own race faces in the emergence of these effects. However, a fundamental limitation in studying the role of experience in human behavior in general and face recognition in particular is isolating the unique contribution of natural face experience to recognition performance on face recognition tasks, independent of other factors. To address this limitation, the present study investigated the effect of experience on the other-race effect, other-age effect, and face inversion effect by systematically varying the training diet of face-trained deep learning algorithms, for which we have complete control over their face experience.

Deep learning models have achieved human-level performance in face recognition tasks for over a decade (Balaban, 2015; Blauch et al., 2021; Taigman et al., 2014). These models are typically trained on millions of face images to reach this level of proficiency, aligning with human performance. This underscores the importance of extensive experience in achieving expert-level face recognition. Following this engineering milestone, cognitive scientists have begun to explore whether face-trained deep learning models exhibit phenomena analogous to those observed in humans, such as the other-race effect and face inversion effect (for reviews see van Dyck & Gruber, 2023; White & Phillips, 2024). Remarkably, the evidence suggests that they do: deep convolutional neural networks (DCNNs) trained predominantly on upright faces from a particular race exhibit superior recognition performance for faces of that race (Dobs et al., 2022, 2023; Tian et al., 2021). Similarly, performance for recognizing children’s faces is notably lower in state-of-the-art DCNNs that are primarily trained on adult faces (Siddiqui et al., 2018), consistent with the other-age effect seen in humans. Finally, face recognition algorithms also show a substantial inversion effect (Tian et al., 2022). Interestingly, both humans and models show a larger inversion effect for within-category discrimination, such as individual faces or birds (Campbell & Tanaka, 2018; Yovel et al., 2023), and a smaller inversion effect for between-category discrimination, such as object categories (Dobs et al., 2022, 2023; Yovel et al., 2023). What remains unknown, however, is how these effects develop as experience accumulates: do they strengthen gradually with increasing training, mirroring the developmental trajectory observed in humans, and is this driven primarily by selective improvements in recognizing own-group upright faces.

The presence of these human-like effects in DCNNs offers a unique opportunity to systematically examine the sole role of experience in the specialization for upright, own-race and own-age faces. We will refer to these faces as upright own-group faces. While findings from such models may not directly translate to human cognitive processes, they allow for controlled investigation of experiential factors and enable comparison with developmental patterns observed in humans. For example, developmental studies show that the increasing magnitude of the face inversion effect with age results primarily from enhanced performance for upright faces, with little improvement for inverted faces (de Heering et al., 2012). If a similar pattern emerges in DCNNs trained on increasing numbers of face images, this suggests that experience alone could underly the effect in humans. Additionally, by varying systematically the number of identities or number of images per identity, we can examine what is the relative contribution of between-identity and within-identity face variability, which cannot be disentangled in human natural experience with faces. Finally, given that the face specialization to upright own-group faces has often been attributed to qualitative differences in face processing (Farah et al., 1995; Mondloch et al., 2002, 2007; Rhodes et al., 2006)—potentially linked to innate mechanisms (Johnson, 2005; Pascalis & Kelly, 2009)—the finding that DCNNs exhibit these effects demonstrates that face specialization can emerge from experience alone.

To this end, in the current study we systematically manipulated the amount of experience provided to DCNNs. Specifically, we trained separate DCNNs on varying total numbers of face identities ([1, 5, 10, 20, 50, 100, 200, 300]) and on different numbers of images per identity ([2, 5, 10, 50, 100, 200, 500, 1000]). For each of these 64 networks, we evaluated performance on a face verification task (i.e., same/different identity matching) involving pairs of faces from the same or different identities that were not included in the training set, for own-race faces, other-race faces, other-age faces in upright and inverted orientation. This design allowed us to isolate the effects of experience on face recognition performance, eliminating confounds associated with human studies, such as innate cognitive differences and the variability and complexity of individual perceptual and social experiences, for which we have neither precise records nor control.

## METHODS

### Model

We used VGG-16 (Simonyan & Zisserman, 2014) as the base model, which we trained on different numbers of face images. We selected this model because it is a well-established widely used architecture that reaches human level performance on face recognition tasks and provides a principled baseline for studying experience-dependent face specialization effects (Abudarham et al., 2021; Blauch et al., 2021; Dobs et al., 2023).

To extract the representations that were generated by the DCNNs, we ran the trained models in evaluation mode on a predefined set of image stimuli. The face images were first aligned using the MTCNN face alignment algorithm (Xiang & Zhu, 2017). Following alignment, the images were normalized with the normalization of (*M* = [0.5, 0.5, 0.5], *SD* = [0.5, 0.5, 0.5]). Then, we extracted the representations at the penultimate, fully connected (fc7) layer. This is the final hidden layer that generates the final representation that is transformed to the output probability layer to determine face identity.

### Train Dataset

We selected face images from the VGGFace2 dataset (Cao et al., 2018) to train our networks. VGGFace2 is a large-scale face recognition dataset developed by the Visual Geometry Group at the University of Oxford. It contains over 3 million images of more than 8,749 individuals, with each individual represented by several hundred images. The images were collected from a variety of sources and were annotated with bounding boxes and labels indicating the identity of the individuals. To allow evaluation of the other-race effect, we used only White adult faces from this dataset.

### Training Protocol

We trained DCNNs on 64 different combinations of identities (2, 5, 10, 50, 100, 200, 500, 1,000) and images per identity (1, 5, 10, 20, 50, 100, 200, 300), creating systematic variation in training experience. To ensure robust estimates for smaller training sets (1–100 identities), where specific image selection could influence results, we trained 30 independent models for each combination. For larger training sets (200–1,000 identities), which are less susceptible to sampling variability, we trained one model per combination. This resulted in 1,224 models total. To examine the other-race effect, all models were trained exclusively on White adult faces and tested on White, Asian, or child faces (see Face Stimuli for Model Performance section below).

### Face Stimuli for Model Performance

The face images used to test model performance were not included in the training set.

Other-race effect: To examine the other-race effect, we selected a subset of 40 same identity and 40 different identity pairs of white faces and a similar number of pairs of Asian faces from the VGGface2 benchmark database. The identities in each pair were from the same sex (half females).

**Figure 1.**
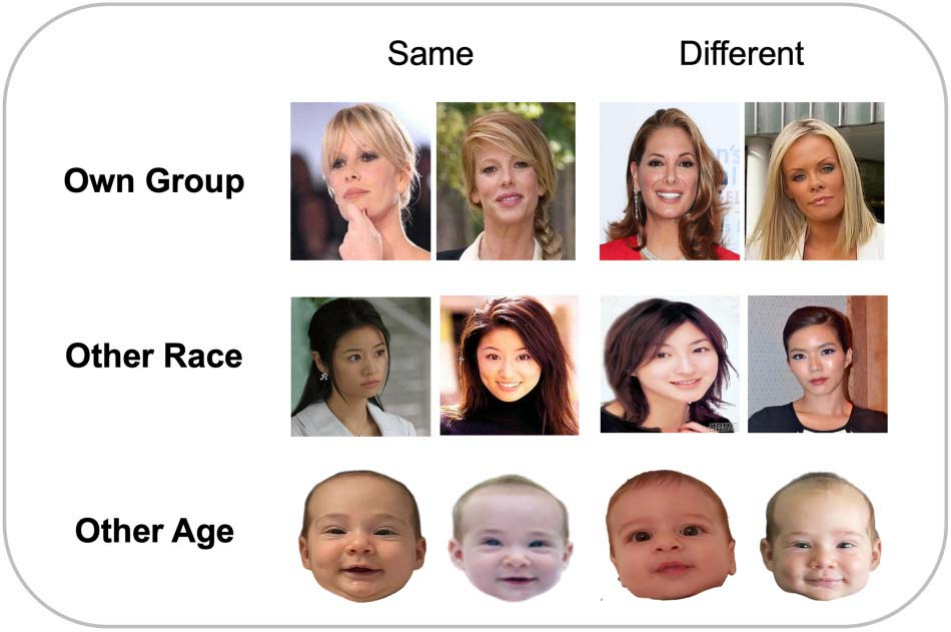
Example of face stimuli used to assess performance level of the different DCNNs for own-group faces taken from the LFW, other-race faces taken from the VGGface2 data set and other-age (infant) faces taken from an in-house infant face dataset. The inversion effect was tested by presenting the same face images upside down.

Face inversion effect: To examine the inversion effect for own-group faces, we used the Labeled Faces in the Wild dataset (LFW; Huang et al., 2008), which includes 6,000 benchmark face pairs. We excluded non-Caucasian identities based on the dataset’s race annotation scores (only scores > 0) and removed identities that overlap with the VGGFace2 dataset. This resulted in 3,506 pairs, which were tested in both upright and inverted orientations (see Figure 1).

Other age effect: To examine the other-age effect we used 44 face images of 4–6 months old infants from an in-house infant data base. For each face identity, we used two different exemplars.

### Performance Measures

We measured the performance of the trained DCNNs on three face verification tasks: own-group, other-race (Asians), and other-age (4–6-month-old babies). In each task half of the pairs were upright, and half were inverted. In all verification tasks, half of the trials were positive pairs, where both images show the same person, and half negative pairs, where the two images show different people. The goal of the verification tasks is to determine if the two images in each pair belong to the same person or not. We assessed the models’ performance by measuring the cosine distance between the embeddings of face pairs. Based on these distances, we computed the Area Under the ROC Curve (AUC), which quantifies the model’s ability to discriminate between same-identity and different-identity pairs across different levels of thresholds. Additionally, we computed accuracy using the optimal threshold determined from the ROC curve. We report AUC values in the main text because they are threshold-independent and the optimal threshold accuracy in the Supplementary materials (Face verification performance using accuracy measures). Results of both measures were similar. The AUC values and optimal-threshold-accuracy scores are available on the Open Science Framework (OSF)—https://osf.io/htmaq/?view_only=4d05a21142af4782abfef2b052912f3d.

### Statistical Analysis

As described in the Training Protocol section, we trained models on 64 different combinations of identities (2, 5, 10, 50, 100, 200, 500, 1,000) and images per identity (1, 5, 10, 20, 50, 100, 200, 300), with 30 independent models for smaller training sets (1–100 identities) and one model for larger sets (200–1,000 identities), resulting in 1,224 models total.

For the ANOVA, we binned models into five experience levels based on total training images: 10^0^, 10^1^, 10^2^, 10^3^, and 10^4^ (e.g., 10 identities × 300 images = 10^3^), resulting in 60, 240, 422, 337 and 160 models per bin, respectively. We excluded the 10^5^ level from analyses due to small sample size (*n* = 5 models vs. *n* ≥ 60 for other levels), though including it yielded similar results. Statistical tests were performed on individual model values, treating each model as an independent observation. Figures show mean AUC across models per experience level, with error bars indicating standard error across different models within each level of experience. Figures with all 64 combinations are presented in Supplementary material (Figures S1–S2).

## RESULTS

We first examined the effect of experience on the magnitude of the other-race effect. To examine this pattern of findings and test them statistically, we grouped the DCNNs based on the total number of training images (calculated as the number of identities multiplied by the number of images per identity) (see Supplementary Figure S1 for results of each of the 64 DCNNs). The models were then categorized into bins according to the integer part of the base-10 logarithm of this total, resulting in five experience levels: 10^0^, 10^1^, 10^2^, 10^3^, and 10^4^, training images (For example, a model trained on 10 identities with 300 images per identity was included in the 10^3^ category).

**Figure 2.**
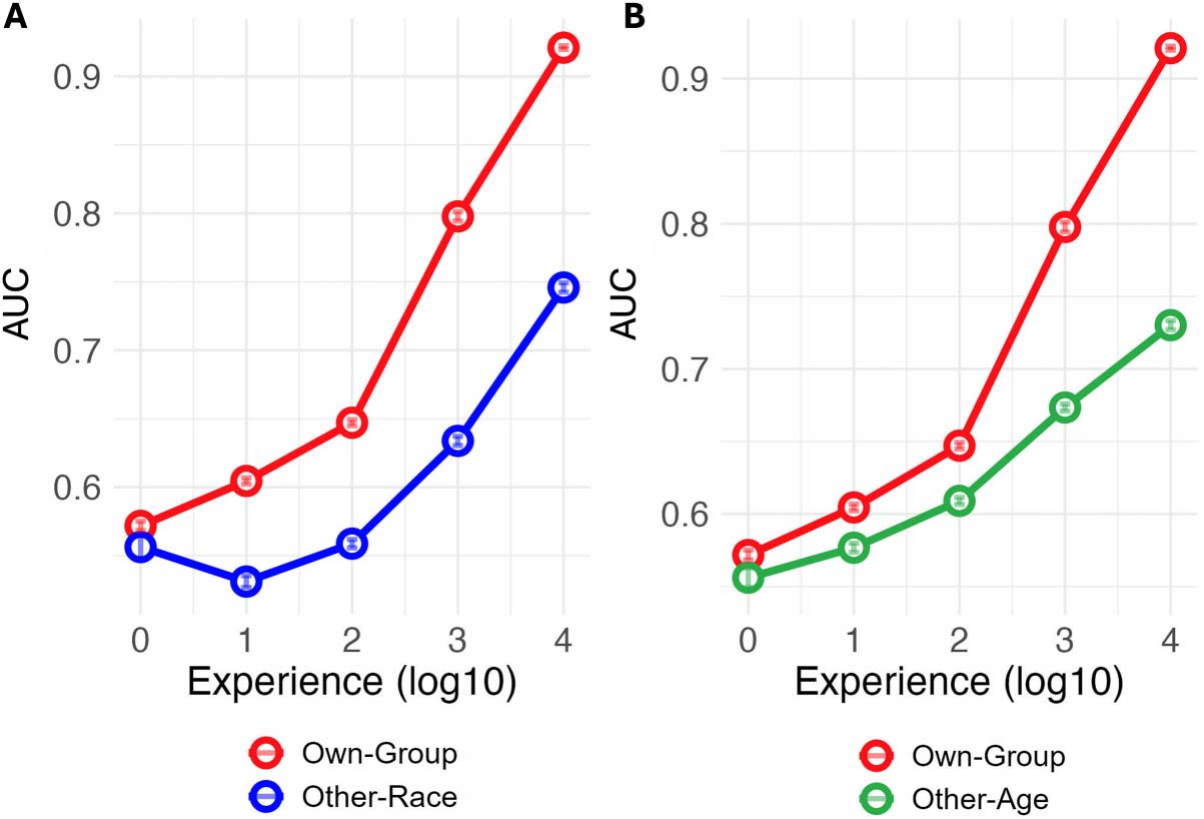
Accuracy values (AUC) values for face verification tasks with upright own-race and other-race faces (left) and upright own-age and other-age faces (right) across different levels of experience with upright own-group faces. Data points represent mean AUC across models within each experience level, with error bars showing standard error (*SE*).

Figure 2 shows that experience with a larger number of own-group upright face images improve performance for all conditions but mostly for own-group faces. To examine this effect statistically, we performed two mixed ANOVAs, one with Face condition (own-group, other-race) as within network factors and Experience Level (10^0^, 10^1^, 10^2^, 10^3^, 10^4^) as a between network factor and another mixed ANOVAs with Face condition (own-group, other-age) as within network factors and Experience Level (10^0^, 10^1^, 10^2^, 10^3^, 10^4^). Both models revealed significant effects of experience level (with other-race: *F*(4, 1214) = 1202, *p* < 0.001, *η*_*p*_^2^ = 0.798; with other-age: *F*(4, 1214) = 1268.63, *p* < 0.001, *η*_*p*_^2^ = 0.807), condition (with other-race: *F*(1, 1214) = 2277.87, *p* < 0.001, *η*_*p*_^2^ = 0.652; with other-age: *F*(1, 1214) = 2167.94, *p* < 0.001, *η*_*p*_^2^ = 0.641), and the interaction (with other-race: *F*(4, 1214) = 186.71, *p* < 0.001, *η*_*p*_^2^ = 0.381; with other-age: *F*(4, 1214) = 477.22, *p* < 0.001, *η*_*p*_^2^ = 0.611).

To further examine the interactions, we performed two sets of contrasts for each level of experiences (Table 1). We first examined the magnitude of the other-race effect and other-age effect for upright faces only. This analysis revealed that performance on upright own-group faces becomes significantly higher than for upright other-race faces from around 10 training images, and higher than for upright other-age faces from around 10 training images. Overall, the contrasts show that as the models become more experienced, they become more specialized for own-group faces.

**Table 1.** Contrast estimates for the AUC differences between own-group and other-group conditions across experience levels. Results are shown separately for race and age models. Standard errors (*SE*), degrees of freedom (*df*), *t*-ratios, and *p*-values are reported (Bonferroni-corrected for 5 comparisons within each model).

**Own vs. Other group Contrasts**	**Experience Level**	**Estimate**	***SE***	***df***	***t*-ratio**	***p*-value**
Own vs. Other race	10^0^	0.016	0.008	1214	1.994	0.232
	**10^1^**	**0.073**	**0.004**	**1214**	**18.934**	**0.0001**
	**10^2^**	**0.088**	**0.003**	**1214**	**30.191**	**<.0001**
	**10^3^**	**0.164**	**0.003**	**1214**	**50.099**	**<.0001**
	**10^4^**	**0.175**	**0.005**	**1214**	**36.875**	**<.0001**
Own vs. Other age	10^0^	0.016	0.006	1214	2.563	0.053
	**10^1^**	**0.028**	**0.003**	**1214**	**9.093**	**<.0001**
	**10^2^**	**0.038**	**0.002**	**1214**	**16.515**	**<.0001**
	**10^3^**	**0.124**	**0.003**	**1214**	**48.258**	**<.0001**
	**10^4^**	**0.191**	**0.004**	**1214**	**51.015**	**<.0001**

**Figure 3.**
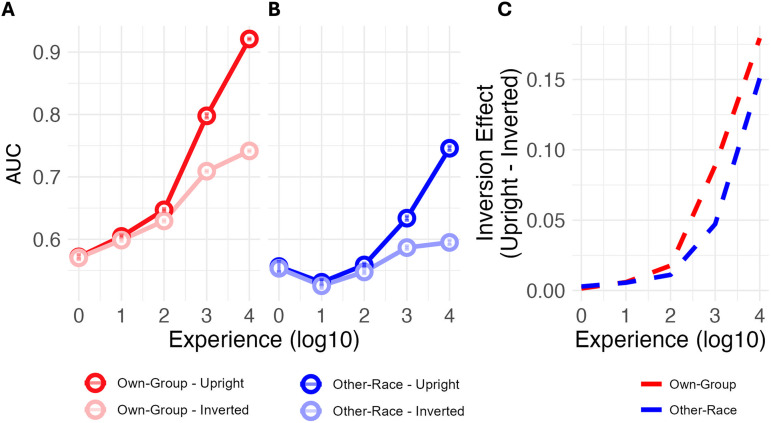
Accuracy values (AUC) values for face verification with upright and inverted faces across varying levels of experience with upright own-race and other-race faces. Left: own-race faces. Middle: other-race faces. Right: magnitude of the inversion effect (difference in AUC between upright and inverted conditions) for own-race and other-race faces. Points show mean AUC across models; error bars indicate SE (left and middle panels only).

We then examined the effect of experience on the face inversion effect for own-group and other-race faces Figure 3A shows that experience improves performance for upright faces whereas performance for inverted faces plateaued at low performance levels and does not improve with further experience. To examine this effect statistically, we performed a mixed ANOVA with Face Orientation (Upright, Inverted) and Face condition (Own-Group, Other-Race) as within network factors and Experience (10^0^, 10^1^, 10^2^, 10^3^, 10^4^) as a between network factor. All main effects and interaction were found significant (*F* > 30, *p* < 0.001; see Supplementary Table S1).

To further examine the magnitude of the inversion effects, we conducted a mixed-ANOVA with Orientation (Upright, Inverted) and Face Condition (Own-Group, Other-Race) as within network factors, and Experience Level (10^0^, 10^1^, 10^2^, 10^3^, 10^4^) as a between networks factor (Full statistics in Supplementary Table S1). Following this, we performed a set of planned contrasts comparing upright and inverted faces in each condition (Own-Group and Other-Race) at each experience level (Figure 3A, B), as well as a comparison of the magnitude of the inversion effect between Own-Group and Other-Race at each experience level (Figure 3C). The contrast analysis showed that the inversion effect for own-group faces as well as for other-race faces emerges following experience with ∼10^2^ faces. Starting from experience level of ∼10^3^ faces the magnitude of the inversion effect in own-group becomes significantly larger than that in other-race faces. Full statistics are presented in Table 2. To control for the influence of upright performance on the inversion effect, we also computed the normalized inversion effect ((upright − inverted)/upright). This analysis showed that own-group faces exhibit a significantly larger normalized inversion effect than other-race faces, starting at the 10^3^ experience level (full statistics in Supplementary Table S4).

**Table 2.** Contrast estimates for the inversion effect in own-group and other-race faces, across different experience levels. Includes standard errors (*SE*), degrees of freedom (*df*), *t*-values, and *p*-values (adjusted using Bonferroni correction for 15 tests). Significant effects are highlighted in **bold**.

**Inversion Effect Contrast**	**Experience Level**	**Estimate**	***SE***	***df***	***t*-ratio**	***p*-value**
Own-group	10^0^	0.001	0.004	1214	0.409	1.0000
	10^1^	0.006	0.002	1214	3.345	0.0127
	**10^2^**	**0.018**	**0.001**	**1214**	**13.254**	**<.0001**
	**10^3^**	**0.089**	**0.001**	**1214**	**59.450**	**<.0001**
	**10^4^**	**0.18**	**0.002**	**1214**	**82.820**	**<.0001**
Other-race	10^0^	0.003	0.007	1214	0.408	1.0000
	10^1^	0.006	0.003	1214	1.610	1.0000
	**10^2^**	**0.011**	**0.003**	**1214**	**4.282**	**<.0001**
	**10^3^**	**0.047**	**0.003**	**1214**	**16.197**	**<.0001**
	**10^4^**	**0.151**	**0.004**	**1214**	**35.712**	**<.0001**
Inversion effect in own-race vs. other-race	10^0^	−0.001	0.007	1214	−0.196	1.0000
	10^1^	0	0.003	1214	0.104	1.0000
	10^2^	0.007	0.003	1214	2.487	0.1951
	**10^3^**	**0.042**	**0.003**	**1214**	**14.135**	**<.0001**
	**10^4^**	**0.029**	**0.004**	**1214**	**6.679**	**<.0001**

**Figure 4.**
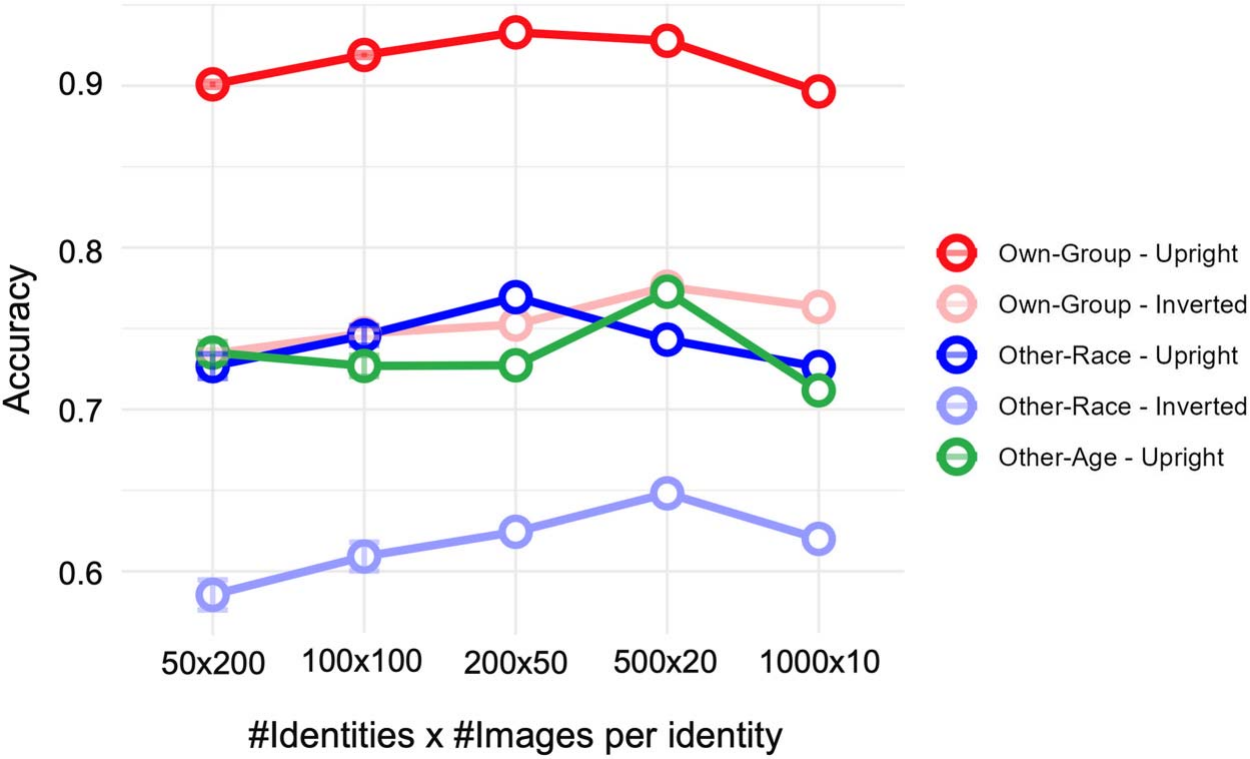
Accuracy values (AUC) values for face verification with upright and inverted faces across own-race, other-race, and other-age conditions. Results are averaged across DCNNs trained on 10,000 images with varying numbers of identities × number images per identity. Error bars show standard error across 30 independently trained models (available only for 50 and 100 identities; 200–1,000 identities used single models; see Training Protocol section).

A final question our training design enables us to answer is whether the number of different identities or the number of images per identity has a larger effect on performance on these tasks. To answer this question, we selected a subset of DCNNs that were trained on the same number of total images—10,000—but different number of identities (50, 100, 200, 500, 1000) and the corresponding images per identity (200, 100, 50, 20, 10). Figure 4 shows performance on these five DCNNs. If images per identity is more important than we expect that performance will be best for the DCNN that was trained on 50 identities × 200 images per identity and worse for a DCNN that was trained on 1000 identities and 10 images per identity. If the number of identities is more important, we would expect the reverse effect. Descriptive evaluation of Figure 4 shows neither of these trends, indicating that both the number of images and number of identities underlies the increased specialization for upright own-group faces.

## DISCUSSION

In this study we used deep learning algorithms to isolate the role of experience in face specialization. Our findings show that increasing the number of upright own-group face images in the training set of DCNNs increases the magnitude of well-established face specialization effects in human face processing - the own-race, own-age, and face inversion effects (Figures 2–3, Supplementary Figure S1). This was primarily due to a steep gain in performance level for upright, own-group faces with modest gains for other-group and inverted faces.

The specialization of face recognition for upright own-group faces is one of the most robust phenomena in human face recognition. This specialization is believed to rely on extensive experience with upright own-group faces and was shown to increase across development (e.g., de Heering et al., 2012; Meinhardt-Injac et al., 2014). Yet multiple innate and experience-based factors were proposed to account for these face effects, including cognitive, social and motivational factors (Bernstein et al., 2007; Farah et al., 1995; Levin, 1996; Meinhardt-Injac et al., 2014; Mondloch et al., 2007; Pascalis & Kelly, 2009; Yin, 1969), making it impossible to isolate the contribution of visual experience with faces to the emergence of face specialization effects. Our findings demonstrate that human-like face specialization can be achieved based on face experience alone in artificial face models.

Notably, our computational approach demonstrates that experience-based learning is sufficient to produce human-like face specialization effects in DCNNs. However, this does not establish that experience operates as the sole mechanism in human development. Important differences exist between DCNN training, which is based on supervised learning with explicit labels via backpropagation and human perceptual learning which likely involves self-supervised, multimodal processes (Vong et al., 2024). Moreover, humans may possess innate predispositions or evolutionary constraints that are not well modelled with DCNNs (Johnson, 2005; Viola Macchi et al., 2004). Our approach therefore provides a computational proof of principle showing that experience-driven learning alone can generate the behavioral signatures of face expertise and reproduce developmental trajectories observed in humans. This demonstrates that experience-based mechanisms are sufficient to produce these effects in artificial systems and provides mechanistic hypotheses for how face specialization might emerge in human development.

The effects of experience that are found in DCNNs mirror some of the effects that are found in humans. First, the increase in the magnitude of the inversion effect as experience with upright faces increases (Figure 3, Supplementary Figure S1) is similar to reports in developmental studies that show that the magnitude of the inversion effect increases across age primarily due to increase in performance for upright faces (de Heering et al., 2012). For the other-age effect, studies have shown better recognition of adult faces than children’s faces in both children and adults (e.g., Cassia et al., 2009; Kuefner et al., 2008), while lab-based or real-life experience with other-age faces abolished the effect (Kuefner et al., 2008; Yovel et al., 2012).

A recent study showed that the other-race effect can be reversed or abolished through exposure to other-race faces—but this reversal was limited after the age of 12, suggesting a potential critical period for face recognition (McKone et al., 2019). The computational approach used in the current study provides a useful framework for modeling the role of such a critical period in the development of the other-race effect. This can be achieved in future studies by introducing other-race faces into the training set at different stages of learning to systematically examine how much exposure is required to reduce or eliminate the effect during early development, when the system is still learning to recognize faces. Once the network reaches a stage of high performance for own-race faces, its parameters may be frozen—simulating a mature system—so that additional exposure to own- or other-race faces no longer alters its internal representations. This may result in continued high performance for familiar own-race faces, but limited improvement for other-race faces, which would require changes to the network’s feature tuning.

In a previous study we used the same DCNNs reported in this study, to examine the role of experience in the generation of view-invariant representations (the ability to generalize across different views of the same identity) and sensitivity to facial features that were shown to be critical for human face recognition (Rosemblaum et al., 2025). The current study complements these findings by showing that increased experience with own-group upright faces is accompanied by category-specific tuning for own-group face identification. Both studies demonstrate that general identity recognition abilities and category-specific tuning emerge together as an outcome of increased experience with faces.

Another advantage of our approach is that it enabled us to assess the relative contribution of the number of identities and number of images per identity. Our findings descriptively show that both the number of identities and the number of images per identity contribute to the generation of the face inversion effect and the other-group effect, indicating that overall training experience, rather than a single component, drives the emergence of these specialization effects. This is consistent with our previous findings that both factors contribute to view invariant representations and sensitivity to human-like critical features for face identity (Rosemblaum et al., 2025). This raises an open empirical question: what is the nature of variability that enables successful performance on face identification task, and whether data augmentation manipulations during training could produce similar effects as training with naturally diverse exemplars in DCNNs.

Our networks were trained exclusively on upright White adult faces, as our baseline condition relative to which we compared Asian faces or inverted faces. However, existing studies demonstrate that these specialization effects generalize to other training categories: networks trained on inverted faces show better performance on inverted than upright faces, and networks trained on Asian faces show better performance on Asian than White faces (Dobs et al., 2023; Tian et al., 2021). While these studies did not systematically vary training experience as we did, they demonstrate that performance improvements occur primarily for the trained category rather than the untrained category, suggesting that the effect that we found is not specific to white upright faces. We therefore expect that the same experience-dependent specialization pattern we observed would emerge for DCNN trained on other face categories. While our study examined how experience supports specialization for upright own-race faces that is evident in humans, such specialization can be abolished by increasing the diversity of the training set in CNNs. Akbari and Dobs (2025) demonstrated that training CNNs on both Asian and White faces substantially reduces the other-race effect through development of an integrated representational space. This may be consistent with studies in humans that are exposed to more heterogeneous categories of races and show reduced or abolished other-race effect. Future studies could use similar approaches to examine how training diversity shapes the emergence and magnitude of specialization effects.

Despite these similarities in the emergence of face specialization between humans and DCNNs, it is important to note that DCNNs and human differ remarkably in their face learning regimes. Our models are trained on static images with high variability, whereas human experience involves dynamic visual information across time. Moreover, DCNNs are trained in batch mode with explicit identity labels and backpropagation—a learning algorithm with no known biological analog (though biologically plausible approximations have been proposed; Lillicrap et al., 2020)—whereas human face learning takes place during critical periods during which input from the environment accumulates gradually through self-supervised mechanisms that extract statistical regularities, likely integrating multimodal information, combined with supervised learning that involves explicit labels. While our findings demonstrate that experience with faces is sufficient to produce specialization effects in a computational system, our approach lacks the developmental trajectory, social context, and direct behavioral comparisons needed to validate mechanistic predictions about human face perception. Our models therefore provide a computational proof of principle that experience-dependent learning can produce face specialization, rather than a mechanistic model of how such learning occurs in the brain.

Moreover, studies that used head-mounted cameras that record infant’s visual diet showed that during the first year of life, infants are exposed to a very small number of identities, but many images per identity (Jayaraman et al., 2015, 2017). Abudarham and Yovel (2025) have recently begun to address these differences with a new model that is trained continuously on a small number of identities with many images per identity, potentially allowing more direct comparison to how infants learn to recognize familiar faces during early development. For example, the phenomenon of perceptual narrowing, where performance for other-race faces is comparable to own race faces up to 6 months of age, but is reduced at 9 months of age, could be tested with models that better capture the type of experience that occurs during this period of time. Using data collected from infant’s head-mounted cameras to train the algorithms can further advance our ability to more closely model human experience (Smith et al., 2015; Vong et al., 2024).

Finally, human face recognition is not limited to visual experience. Each face is typically associated with a unique voice, smell, and other social and conceptual knowledge. This multimodal information may enable self-supervised learning across modalities. Future studies need to assess the contribution of multimodal information to face recognition. Thus, our findings do not show that these factors or others (like social and motivational factors) do not contribute to face recognition in humans, but that in principle, visual experience with faces alone can produce similar face specialization effects.

To summarize, we systematically manipulated visual experience by training deep learning algorithms on varying combinations of identities and images per identity. This enabled us to isolate the effect of experience on face specialization. Our findings show that increasing training with upright own-group faces increases the system’s specialization to the type of faces it is trained to identify, while performance for other-group or inverted faces remains low. These findings mirror effects shown in humans across development, suggesting that experience alone may shape human face specialization. Future computational models should close the gap between humans and face-trained deep neural networks by using self-supervised, multimodal continual learning training methods.

## FUNDING INFORMATION

This research was supported by a grant from the Israel Science Foundation (ISF Grant 917/21 and 1321/24 awarded to G.Y.).

## AUTHOR CONTRIBUTIONS

N.G.: Conceptualization: Equal; Formal analysis: Lead; Methodology: Equal; Visualization: Lead; Writing – original draft: Equal; Writing – review & editing: Equal. M.R.: Conceptualization: Equal; Methodology: Equal. G.Y.: Conceptualization: Equal; Funding acquisition: Lead; Supervision: Lead; Writing – original draft: Equal; Writing – review & editing: Equal.

## DATA AVAILABILITY STATEMENT

The AUC values and optimal-threshold accuracy scores analyzed in this study are available on the Open Science Framework (OSF) at https://osf.io/htmaq/?view_only=4d05a21142af4782abfef2b052912f3d. Training code and analysis scripts are available upon request from the corresponding author.

## Supplementary Material


