# Supplementary material for: Experience Alone Can Generate Human Face Specialization: Evidence From Deep Learning Models
Source: Open Mind (Camb). 2026 Jul 7;10:908–22. doi: 10.1162/OPMI.a.363 (PMC13379305; doi:10.1162/OPMI.a.363)
Supplement: Supplementary file 1 [file opmi-10-908-s001.docx]

**Supplementary Material**

**Experience Alone Can Generate Human Face Specialization:**

**Evidence from Deep Learning models**

**The effect of visual experience on the other-group effect and face inversion effect**

The performance of each of the 64 DNNs included in our analysis is displayed in Figure S1. Each DNN was trained on a different combination of the number of identities and the number of images per identity, allowing us to systematically vary within-identity and between-identity variability in the total amount of visual experience. Figure S1 shows the average performance on a verification task for each type of test images (own-group, other-race, other-age, see example images in Figure 1) in both orientations (upright and inverted), for each experience condition (defined by the combination of number of identities (color) and number of images per identity (x-axis). The smaller DNNs, which included 1-100 identities, were trained on 30 different samples of upright own group face images. Performance was computed for each of them and averaged across the DNNs. The larger DNNs (200, 500, 1000 identities) were trained with one set of images.

**
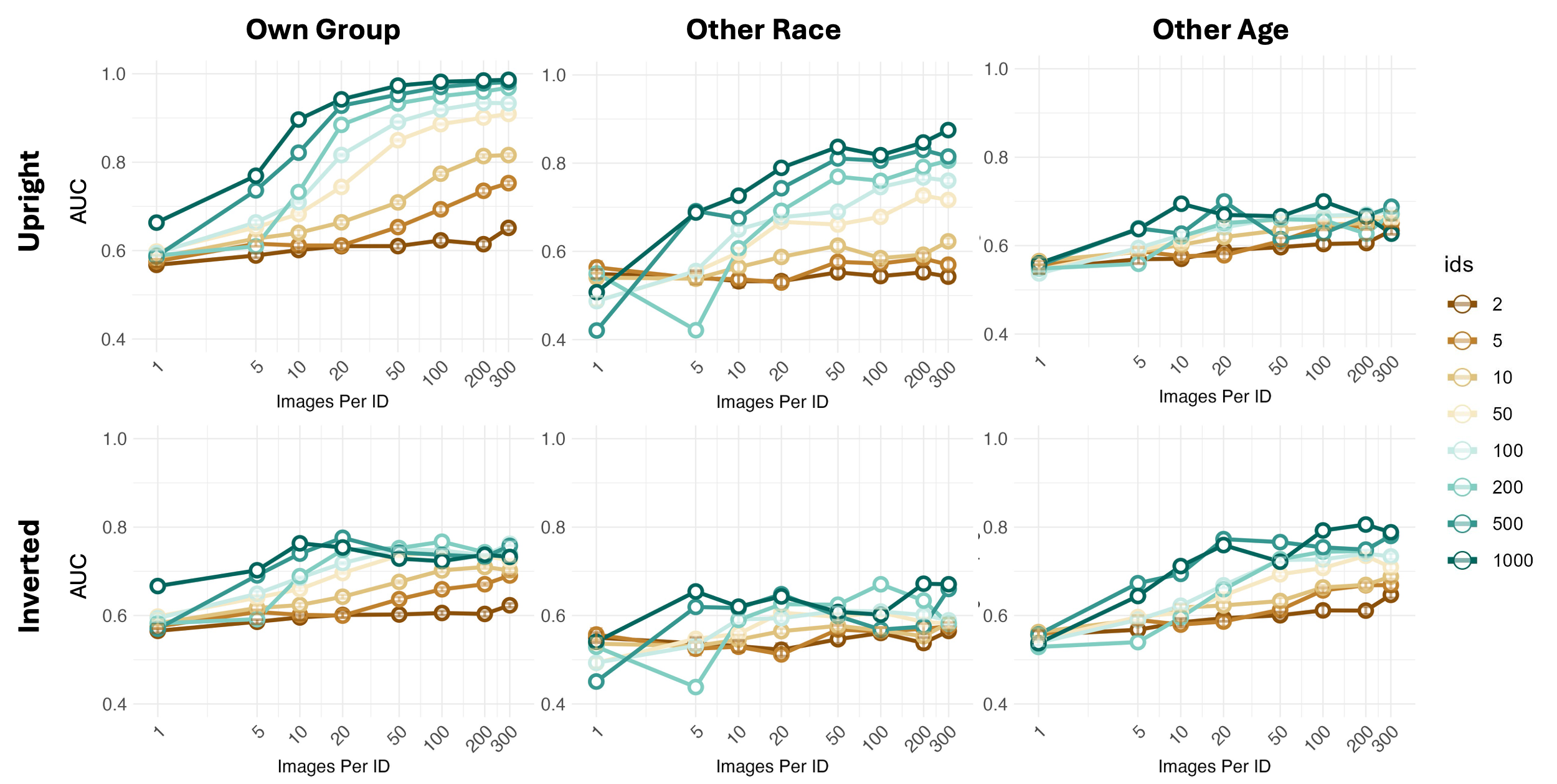
**

Figure S1: Average AUC values across all face verification tasks, shown for each model experience condition (defined by the number of identities and number of images per identity). Performance is presented separately for own-group faces (left), other-race faces (middle), and other-age faces (right), for both upright faces (top row) and inverted faces (bottom row). Error bars are the standard errors of performance level of the DNNs that were trained on 30 different samples of upright own-race face images.

**Analysis of variance of the effects of visual experience, condition and orientation**

Table S2 displays the full statistics of a mixed-ANOVA for AUC values with Orientation (Upright, Inverted) and Face Condition (Own-Group, Other-Race) as within network factors, and Experience Level (10, 10^1^, 10^2^, 10^3^, 10^4^) as a between networks factor. All main effects and interactions are statistically significant.

Table S1: Results of a mixed ANOVA with Face Orientation (Upright, Inverted) and Face Condition (Own Group, Other race) as within network factors and Experience Level (${10}^{0}$, ${10}^{1}$*,* ${10}^{2}$*,* ${10}^{3}$*,* ${10}^{4}$) as a between network factor.

| **Effect** | **df** | **F** | $\eta_{p}^{2}$ | **p-value** |
| --- | --- | --- | --- | --- |
| Experience Level | 4, 1214 | 873.77 | .742 | < .001 |
| Condition | 1, 1214 | 2728.23 | .692 | < .001 |
| Experience Level: Condition | 4, 1214 | 181.06 | .374 | < .001 |
| Orientation | 1, 1214 | 1871.96 | .607 | < .001 |
| Experience Level: Orientation | 4, 1214 | 781.46 | .720 | < .001 |
| Condition: Orientation | 1, 1214 | 60.50 | .047 | < .001 |
| Experience Level: Condition: Orientation | 4, 1214 | 30.64 | .092 | < .001 |

**Face verification performance using optimal-threshold accuracy**

The main text reports AUC values as the primary performance measure. Here we present corresponding accuracy results using optimal thresholds determined from each model's ROC curve.

***The effect of visual experience on the other-group effect and face inversion effect – using accuracy***

Here we present the corresponding results using threshold-based accuracy. Figure S2 shows average optimal-threshold accuracy for each test condition (own-group, other-race, other-age) in both orientations (upright and inverted) across all 64 DNNs, organized by number of identities (color) and images per identity (x-axis). The pattern of results mirrors those observed with AUC measures (Figure S1).

***
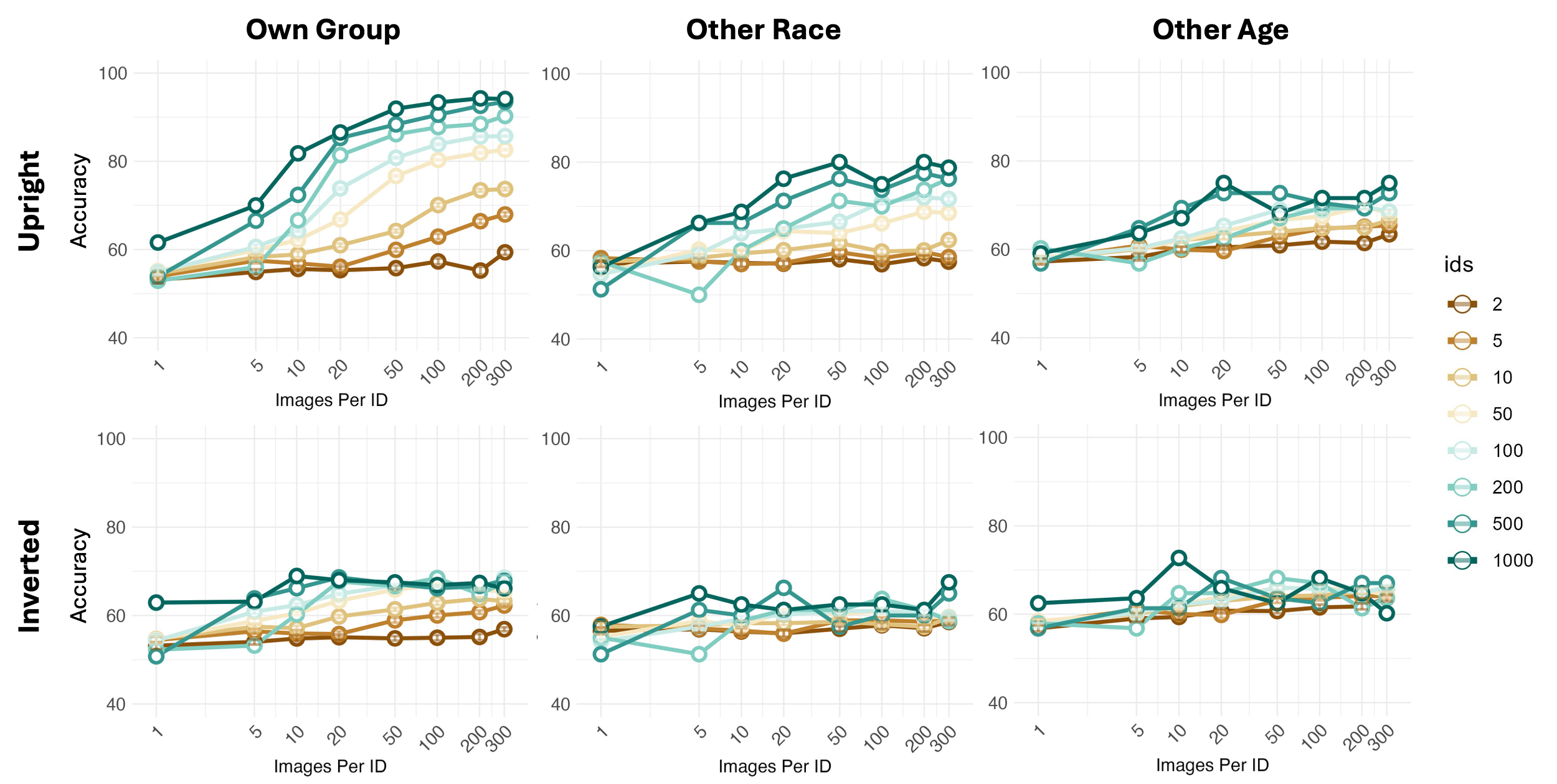
***

Figure S2: Average accuracy across all face verification tasks, shown for each model experience condition (defined by the number of identities and number of images per identity). Performance is presented separately for own-group faces (left), other-race faces (middle), and other-age faces (right), for both upright faces (top row) and inverted faces (bottom row).

***The effect of experience on the magnitude of the other-race and other-age effects – using optimal-threshold accuracy***

To examine the effect of experience on recognition performance, we performed two mixed ANOVAs with Face condition (own-group vs. other-race; own-group vs. other-age) as within-network factors and Experience Level (10⁰, 10¹, 10², 10³, 10⁴) as a between-network factor, using accuracy as the dependent variable.

Both models revealed significant effects of experience level (with other-race: F(4,1214) = 1170.57, p < 0.001, $\eta_{p}^{2}$ = 0.794; with other-age: F(4,1214) = 1164.48, p < 0.001, $\eta_{p}^{2}$ = 0.793), condition (with other-race: F(1, 1214) = 454.73, p < 0.001, $\eta_{p}^{2}$ = 0.273; with other-age: F(1, 1214) = 221.47, p < 0.001, $\eta_{p}^{2}$ = 0.154), and the interaction (with other-race: F(4,1214) = 443.20, p < 0.001, $\eta_{p}^{2}$ = 0.594; with other-age: F(4,1214) = 685.91, p < 0.001, $\eta_{p}^{2}$ = 0.693).


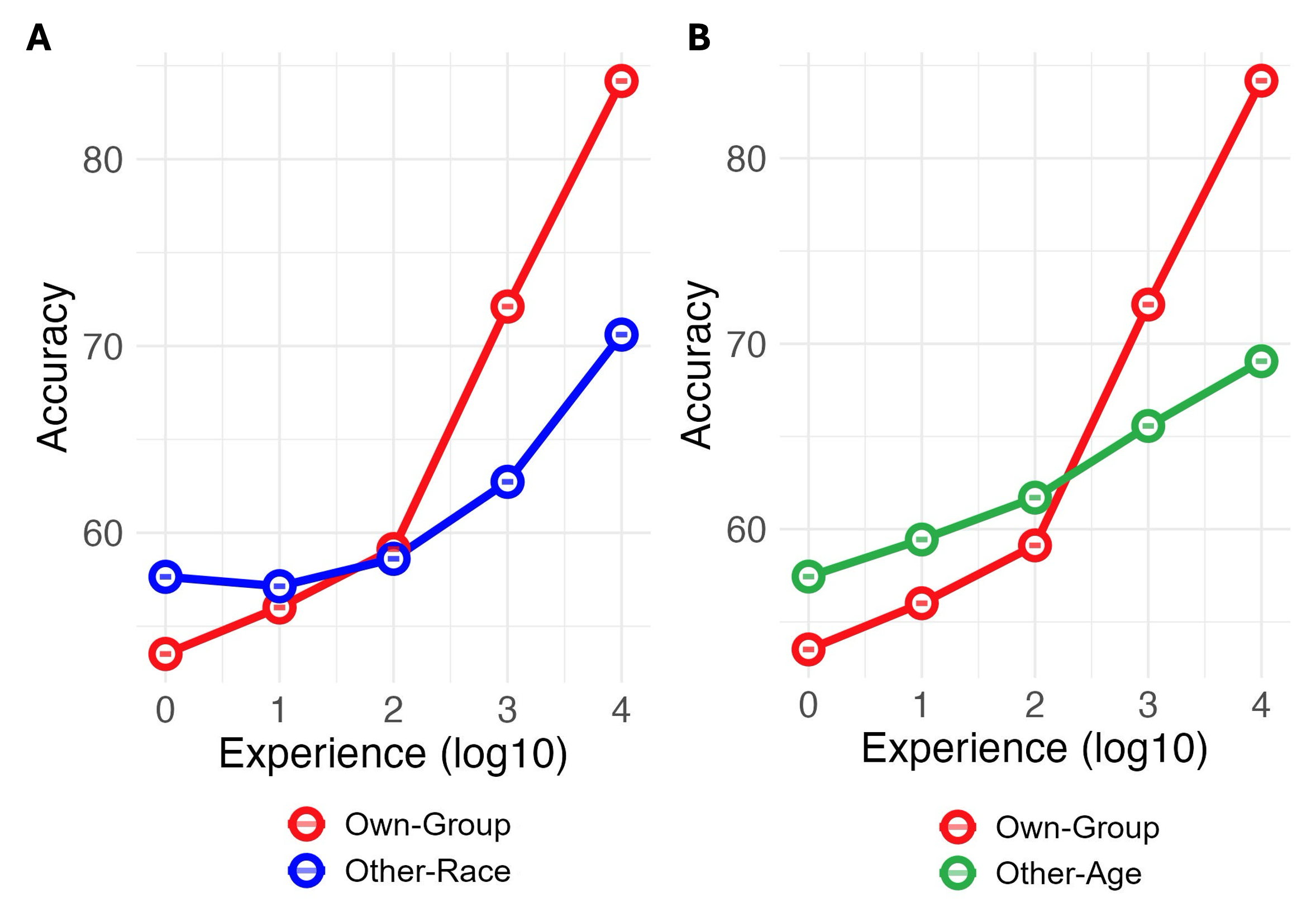


Figure S3: Accuracy values for face verification tasks with upright own-race and other-race faces (left) and upright own-age and other-age faces (right) across different levels of experience with upright own-group faces. Data points represent mean accuracy across models within each experience level, with error bars showing standard error (SE).

To examine at which level of experience the own-group advantage emerged, we examined pairwise contrasts at each level (Table S2). For both other-race and other-age comparisons, models with experience ≥$10^{3}$ showed significantly better performance for own-group faces (positive contrast estimates, all p < 0.001) using accuracy measures.

Table S2: Contrast estimates for the accuracy difference between own-group and other-group conditions (race and age) across experience levels. Results are shown separately for race and age models. Standard errors (SE), degrees of freedom (df), t-ratios, and p-values are reported (Bonferroni-corrected for 5 comparisons within each model).

| Own vs Other group Contrasts | Experience Level | Estimate | SE | df | t-ratio | p-value |
| --- | --- | --- | --- | --- | --- | --- |
| Own vs. Other race | ${10}^{0}$ | -0.041 | 0.006 | 1214 | -6.753 | <0.001 |
|  | ${10}^{1}$ | -0.011 | 0.003 | 1214 | -3.716 | 0.001 |
|  | ${10}^{2}$ | 0.005 | 0.002 | 1214 | 2.23 | 0.13 |
|  | $\boldsymbol{10}^{\boldsymbol{3}}$ | **0.094** | **0.003** | **1214** | **36.326** | **<.0001** |
|  | $\boldsymbol{10}^{\boldsymbol{4}}$ | **0.136** | **0.004** | **1214** | **36.226** | **<.0001** |
| Own vs. Other age | ${10}^{0}$ | -0.039 | 0.006 | 1214 | -6.953 | <.0001 |
|  | ${10}^{1}$ | -0.344 | 0.003 | 1214 | -12.167 | <.0001 |
|  | ${10}^{2}$ | -0.026 | 0.002 | 1214 | -12.085 | <.0001 |
|  | $\boldsymbol{10}^{\boldsymbol{3}}$ | **0.065** | **0.002** | **1214** | **27.442** | **<.0001** |
|  | $\boldsymbol{10}^{\boldsymbol{4}}$ | **0.151** | **0.003** | **1214** | **43.719** | **<.0001** |

***The effect of experience on the face inversion effect for own-group and other-race faces – using optimal-threshold accuracy***

We examined the effect of experience on the face inversion effect for own-group and other-race faces using optimal-threshold accuracy as the performance metric (Supplementary Figure S3). To examine this effect statistically, we performed a mixed ANOVA with Face Orientation (Upright, Inverted) and Face Condition (Own-Group, Other-Race) as within-network factors and Experience Level ($10⁰, 10¹, 10^{2}, 10^{3}, 10⁴$) as a between-network factor. All main effects and interactions were significant (all F > 98, p < 0.001; see Supplementary Table S3 for full statistics).

To further examine the magnitude of the inversion effects, we performed planned contrasts comparing upright and inverted faces in each condition (Own-Group and Other-Race) at each experience level, as well as comparing the magnitude of the inversion effect between Own-Group and Other-Race at each experience level (Supplementary Table S4). The contrast analysis showed that the inversion effect for own-group and other-race faces emerges following experience with ~$10^{2}$ faces. Starting from experience level ~10³, the magnitude of the inversion effect for own-group faces becomes significantly larger than that for other-race faces.

To control for the influence of upright performance on the inversion effect, we also computed the normalized inversion effect ((upright – inverted)/upright). This analysis is described in the next supplementary section below.


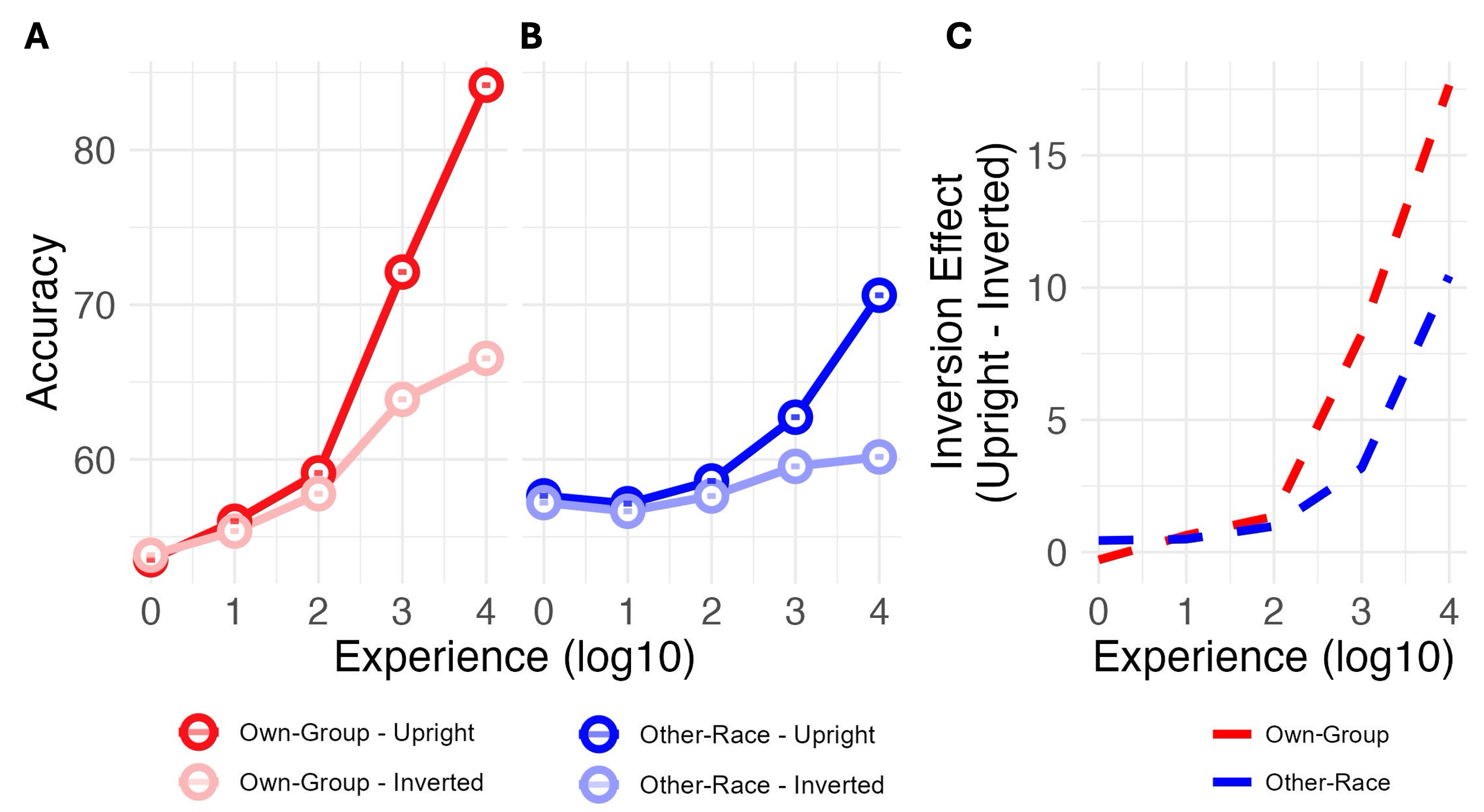


Figure S3: Accuracy values for face verification with upright and inverted faces across varying levels of experience with upright own-race and other-race faces. Left: own-race faces. Middle: other-race faces. Right: magnitude of the inversion effect (difference in Accuracy between upright and inverted conditions) for own-race and other-race faces. Points show mean Accuracy across models; error bars indicate SE (left and middle panels only).

Table S3: Table S3: Accuracy values for face verification with upright and inverted faces across varying levels of experience with upright own-race and other-race faces. Left: own-race faces. Middle: other-race faces. Right: magnitude of the inversion effect (difference in AUC between upright and inverted conditions) for own-race and other-race faces. Points show mean AUC across models; error bars indicate SE (left and middle panels only).

| **Effect** | **df** | **F** | $\eta_{p}^{2}$ | **p-value** |
| --- | --- | --- | --- | --- |
| Experience Level | 4, 1214 | 823.45 | .731 | < .001 |
| Condition | 1, 1214 | 331.52 | .215 | < .001 |
| Experience Level: Condition | 4, 1214 | 412.6 | .576 | < .001 |
| Orientation | 1, 1214 | 1769.01 | .593 | < .001 |
| Experience Level: Orientation | 4, 1214 | 750.43 | .712 | < .001 |
| Condition: Orientation | 1, 1214 | 182.86 | .131 | < .001 |
| Experience Level: Condition: Orientation | 4, 1214 | 98.24 | .245 | < .001 |

| Inversion effect contrast | Experience Level | Estimate | SE | df | t-ratio | p-value |
| --- | --- | --- | --- | --- | --- | --- |
| Own-group | ${10}^{0}$ | -0.003 | 0.005 | 1214 | -0.636 | 1.0000 |
|  | ${10}^{1}$ | 0.006 | 0.002 | 1214 | 2.733 | 0.096 |
|  | $\boldsymbol{10}^{\boldsymbol{2}}$ | **0.013** | **0.002** | **1214** | **7.691** | **<.0001** |
|  | $\boldsymbol{10}^{\boldsymbol{3}}$ | **0.082** | **0.002** | **1214** | **42.050** | **<.0001** |
|  | $\boldsymbol{10}^{\boldsymbol{4}}$ | **0.177** | **0.003** | **1214** | **62.136** | **<.0001** |
| Other-race | ${10}^{0}$ | 0.004 | 0.005 | 1214 | 0.861 | 1.0000 |
|  | ${10}^{1}$ | 0.005 | 0.003 | 1214 | 1.926 | 0.815 |
|  | $\boldsymbol{10}^{\boldsymbol{2}}$ | **0.010** | **0.002** | **1214** | **5.084** | **<.0001** |
|  | $\boldsymbol{10}^{\boldsymbol{3}}$ | **0.032** | **0.002** | **1214** | **14.820** | **<.0001** |
|  | $\boldsymbol{10}^{\boldsymbol{4}}$ | **0.104** | **0.003** | **1214** | **33.556** | **<.0001** |
| Inversion effect in own-race vs. other-race | ${10}^{0}$ | -0.007 | 0.006 | 1214 | -1.148 | 1.0000 |
|  | ${10}^{1}$ | 0.001 | 0.003 | 1214 | 0.452 | 1.0000 |
|  | ${10}^{2}$ | 0.004 | 0.002 | 1214 | 1.540 | 1.0000 |
|  | $\boldsymbol{10}^{\boldsymbol{3}}$ | **0.051** | **0.003** | **1214** | **18.755** | **<.0001** |
|  | $\boldsymbol{10}^{\boldsymbol{4}}$ | **0.072** | **0.004** | **1214** | **18.433** | **<.0001** |

Table S4: Contrast estimates for the inversion effect in own-group and other-race faces, across different experience levels based on an optimal-threshold accuracy measure. Includes standard errors (SE), degrees of freedom (df), t-values, and p-values (adjusted using Bonferroni correction for 15 tests). Significant effects are highlighted in bold.

***Relative contribution of number of identities versus images per identity – using optimal-threshold accuracy***

Our training design enabled us to examine whether the number of different identities or the number of images per identity has a larger effect on performance measured by accuracy. We selected DNNs trained on 10,000 total images but with varying numbers of identities (50, 100, 200, 500, 1000) and corresponding images per identity (200, 100, 50, 20, 10).

**
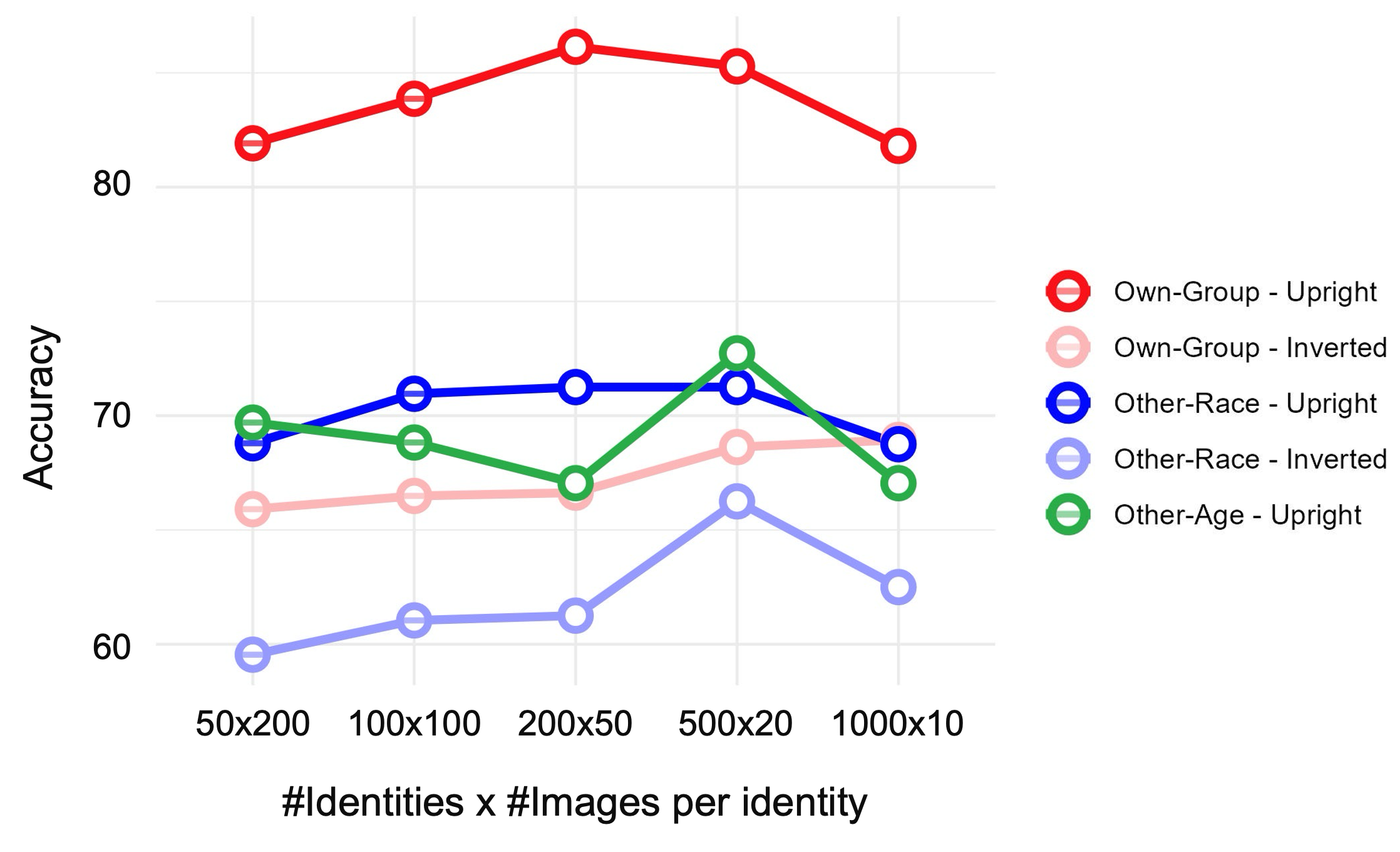
**Figure S4 shows performance on these five DNNs using accuracy measures. The results show neither clear trend, indicating that both factors contribute to the development of specialization for upright own-group faces. This pattern shows the same pattern as the AUC presented in the main text.

Figure S4: Accuracy values for face verification with upright and inverted faces across own-race, other-race, and other-age conditions. Results are averaged across DCNNs trained on 10,000 images with varying identity counts (i.e., different numbers of images per identity). Error bars show standard error across 30 independently trained models (available only for 50 and 100 identities; 200-1000 identities used single models; see Training protocol (2.3)).

**Normalized inversion effect analysis**

In addition to the inversion effect reported in the main text, we also computed a normalized inversion effect ((upright – inverted) / upright). Here we describe the full statistics of the normalized inversion effect differences between own-group and other-race faces in the AUC and optimal-threshold accuracy measures. We used paired t-tests to evaluate the difference between own-group and other-race at each experience level. Significant p-values are those under 0.01 (corrected for 5 comparisons). Both measures show that there are no differences between own-group and other-race faces in small training datasets. Larger training datasets showed higher normalized inversion effects in own-group compared to other-race faces. For AUC, this difference was significant at experience level $10^{3}$, while for accuracy, it was significant at both $10^{3}$ and $10⁴$ experience levels.

Table S5. Comparison of normalized inversion effects between own-group and other-race faces across experience levels – Normalized inversion effect calculated as (upright – inverted) / upright. Paired t-tests compared own-group versus other-race at each experience level. Bold values indicate p < 0.01 (Bonferroni-corrected threshold for 5 comparisons).

| Inversion effect in own-race vs. other-race | Experience Level | Mean Own-Group | Mean Other-Race | Diff | t-value | p-value |
| --- | --- | --- | --- | --- | --- | --- |
| AUC | ${10}^{0}$ | 0.002 | 0.005 | -0.003 | -0.52 | 0.6 |
|  | ${10}^{1}$ | 0.009 | 0.008 | -0.001 | 0.25 | 0.81 |
|  | ${10}^{2}$ | 0.026 | 0.015 | 0.011 | 2.59 | 0.01 |
|  | $\boldsymbol{10}^{\boldsymbol{3}}$ | **0.108** | **0.067** | **0.041** | **6.99** | **<0.001** |
|  | ${10}^{4}$ | 0.195 | 0.2 | -0.005 | -0.81 | 0.42 |
| ACC | ${10}^{0}$ | -0.011 | 0.007 | -0.018 | -1.24 | 0.22 |
|  | ${10}^{1}$ | 0.01 | 0.008 | 0.002 | 0.45 | 0.65 |
|  | ${10}^{2}$ | 0.021 | 0.014 | 0.007 | 1.75 | 0.08 |
|  | $\boldsymbol{10}^{\boldsymbol{3}}$ | **0.11** | **0.047** | **0.063** | **13.8** | **<.0001** |
|  | $\boldsymbol{10}^{\boldsymbol{4}}$ | **0.209** | **0.146** | **0.064** | **12** | **<.0001** |
